# Knowledge translation on dementia: a cluster randomized trial to compare a blended learning approach with a "classical" advanced training in GP quality circles

**DOI:** 10.1186/1472-6963-7-92

**Published:** 2007-06-22

**Authors:** Horst C Vollmar, Martin E Butzlaff, Rolf Lefering, Monika A Rieger

**Affiliations:** 1 Competence Center for General Practice and Outpatients' Health Care, Witten/Herdecke University, Alfred-Herrhausen-Str. 50, D-58448 Witten, Germany; 2 Faculty of Medicine, Witten/Herdecke University, Alfred-Herrhausen-Str. 50, D-58448 Witten, Germany; 3 IFOM – Institute for Research in Operative Medicine, University of Witten/Herdecke Ostmerheimer Str. 200, D-51109 Cologne, Germany

## Abstract

**Background:**

Thus far important findings regarding the dementia syndrome have been implemented into patients' medical care only inadequately. A professional training accounting for both, general practitioners' (GP) needs and learning preferences as well as care-relevant aspects could be a major step towards improving medical care. In the WIDA-study, entitled "Knowledge translation on dementia in general practice" two different training concepts are developed, implemented and evaluated. Both concepts are building on an evidence-based, GP-related dementia guideline and communicate the guideline's essential insights.

**Methods/Design:**

Both development and implementation emphasize a procedure that is well-accepted in practice and, thus, can achieve a high degree of external validity. This is particularly guaranteed through the preparation of training material and the fact that general practitioners' quality circles (QC) are addressed. The evaluation of the two training concepts is carried out by comparing two groups of GPs to which several quality circles have been randomly assigned. The primary outcome is the GPs' knowledge gain. Secondary outcomes are designed to indicate the training's potential effects on the GPs' practical actions. In the first training concept (study arm A) GPs participate in a structured case discussion prepared for by internet-based learning material ("blended-learning" approach). The second training concept (study arm B) relies on frontal medical training in the form of a slide presentation and follow-up discussion ("classical" approach).

**Discussion:**

This paper presents the outline of a cluster-randomized trial which has been peer reviewed and support by a national funding organization – Federal Ministry of Education and Research (BMBF) – and is approved by an ethics commission. The data collection has started in August 2006 and the results will be published independently of the study's outcome.

**Trial Registration:**

Current Controlled Trials [ISRCTN36550981]

## Background

Dementia is a highly prevalent syndrome in industrial nations. In Germany, due to demographic developments, an increase of dementia patients can be expected. According to estimations, only 20% of all persons with dementia are adequately treated in Germany [1-4]. It seems that shortcomings in professional education and training, particularly among general practitioners (GPs) are one of the reasons for this deficiency in medical care [2-5]. Findings indicate that GPs frequently feel awkward about having to communicate with patients and their relatives about diagnosis "dementia" and therefore do not make a serious attempt at diagnosis [6-9]. In order to overcome these barriers, it is necessary to develop and evaluate appropriate instruments suited to sustainable supporting GPs' knowledge and competence in dealing with the dementia syndrome and therefore to improving medical care.

For this purpose, two training concepts have been developed in the WIDA-study which will be implemented in GP quality circles:

1. In study concept A, GPs participate in a structured case discussion in the context of quality circles. The structured discussion is prepared by the GPs in advance via internet-based learning material ("blended-learning" approach).

2. In study concept B, participants are instructed in a classical way with an oral presentation. A structured discussion of the subject matters follows the presentation of the instructor ("classical" approach).

Both training concepts communicate the essential points of an evidence-based guideline on diagnosis and treatment of dementia focusing on GPs. The effects of the two concepts are compared in a cluster randomized controlled study. In addition possible barriers of implementation will be identified.

### Dementia

The term *dementia *describes an etiologically heterogeneous clinical syndrome characterized by the loss of intellectual and mnestic abilities. Depending on etiology, dementia-related syndromes can be found in any stage of life. However, they generally show a continuously increasing prevalence in the 60+ age groups. While for those aged 65–69 incidence is below 2%, there is a rise to a 10–17% incidence for those aged 80–84; for those over 90 the incidence reaches 30% [10].

The most frequently occurring dementia-related forms are

• the so-called *degenerative forms*, particularly dementias of the Alzheimer's type,

• followed by *vascular dementias *due to ischemically caused destruction of brain tissue and

• *mixed degenerative-vascular forms *of dementia.

Alzheimer's dementia tends to display no physical or neurological symptoms in the first years. Cognitive malfunctions generally manifest themselves in the form of a progressive reduction of short-term memory, orientation ability and word-finding difficulties. Complex daily tasks can no longer be accomplished. Typically, there is a creeping onset of the disease, which initially proceeds slowly. The intermediary stage is characterized by a disfunctioning of the long-term memory and the patients' ability to think clearly. Patients are no longer able to work in their vocation and cannot manage their household. Apart from this, non-cognitive disorders such as agitation, irritability, emotional instability or aggression, and even apathy and depression can occur. Moreover, a urinary incontinence (aconuresis) can frequently be detected. The late stage of Alzheimer's disease is characterized by profound intellectual degeneration and comprehensive need for nursing care due to symptoms such as complete urinary and anal incontinence, impaired movement, inability to control posture, cerebral convulsions, and swallowing disfunctionality. Dementia patients normally spend the late stages of the disease in a nursing home.

Further facts about the dementia syndrome:

• Presently there are about one million persons suffering from dementia in Germany [5,11,12]; among them approximately 700,000 suffer from Alzheimer's disease [11,12]. More than 200,000 new cases of dementia are reported each year, 125,000 of which can be attributed to Alzheimer's disease. Projections which consider demographic developments predict that the number of dementia patients will increase to more than 2 millions by 2050 in Germany [11,12].

• As a general rule, dementia displays a chronically progressive development: beginning with the first disease-related symptoms, the average survival period is eight to nine years. According to more recent studies, the median survival time following clinical diagnosis is 4.2 years for men and 5.7 years for women [13,14]. A Canadian study even indicates a median survival time of just 3.3 years after clinical diagnosis [15].

• Dementia has far-reaching consequences for both the patient and his or her relatives: Apart from the loss of autonomy and an increasing need for nursing care, dementia patients frequently suffer from malnutrition or undernourishment and an increased susceptibility to infection. Persons with dementia also display a greater risk of contracting pneumonia, seizure disorders, or pressure ulcers [5,16]. Furthermore, there is a heightened risk of accident as well as an increased risk of becoming a victim of crime [5].

• In most cases, negative consequences for the social environment surface quite rapidly; caregivers and relatives of persons with dementia often suffer from a high degree of physical and mental stress [5,17].

• Dementia is one of the most costly diseases [17,18]. There are only a few current studies on the costs of dementia treatment in Germany. However, their results are comparable to those found in international studies [11,18]. For the year 2002, the Federal Office of Statistics assumed that costs amounted to as much as € 5.6 ($ 7.3) billion, with 60% of the total costs incurred for in-patient or partially stationary care [19].

• There are many reasons for the above-mentioned problems in the treatment of dementia. [2,5,20]:

∘ Either the acknowledgement of dementia is considered taboo or dementia is classified as a form of aging.

∘ Knowledge of diagnostic procedures and adequate treatment is deficient.

∘ Diagnosis mostly occurs in the advanced stages of the disease.

∘ Many nursing homes are not prepared to treat individuals with dementia and/or have a shortage of staff.

∘ People with dementia naturally have a hard time articulating their discomfort and wishes.

∘ A comprehensive therapeutic approach does not exist.

∘ Newly-developed drugs are too expensive (limited health care budgets); the effectiveness is controversially judged and therefore they are only reservedly prescribed.

∘ *Further education and training programs in the medical profession are inadequate*.

### Perspective of General Practice

Dementia is a syndrome that typically occurs in general medicine even though the setting of general practice is only rarely considered in the literature [6-9,21-33]. Wagner and Abholz stated:" The medical and psychosocial care of people with dementia lies largely in the hands of GPs. The GP knows the patient, his or her relatives, and the social setting. Therefore, s/he is in the best position to notice even slight changes of intellectual performance"[7]. The same publication points out that GPs – due to the lack of therapeutic consequences – do not seriously wish to diagnose the illness. Moreover, there is evidence that GPs feel awkward about having to talk with patients about such an emotionally charged diagnosis [6-8,33]. A study carried out in Wales and England with more than 1,000 GPs found that only 52% of all respondents considered early diagnosis to be useful [34]. A 1992 Australian study observed only marginal knowledge of dementia on the part of primary care practitioners [35]. In Germany, GPs frequently diagnose dementia only when the need of nursing care already exists [36].

A summary of various studies shows that GPs either lack knowledge and professional skills in the treatment of dementia or do not make use of their existing knowledge when treating their patients.

### Guidelines

Field and Lohr define clinical practice guidelines (CPGs) as "systematically developed decision support regarding the adequate medical *modus operandi *for special health-related problems"[37]. Moreover, the following aspects are relevant [38]:

• Guidelines present the consensus reached by several experts from diverse faculties and working groups on certain medical approaches (if possible by considering the patients' view).

• Guidelines are scientifically founded recommendations for action.

• Methodological instruments for creating guidelines are (among others): consensus conferences, Delphi analyses, therapeutic studies, and meta-analyses.

• Guidelines are orientation aids in the sense of "guidelines for action and decision making". In some cases divergence from the guideline is possible or even essential.

• Guidelines are regularly checked and updated if necessary.

Evidence-based medical guidelines are considered to be the central implementation tool for evidence-based medicine (EbM). In this capacity they can also serve as a basis for further professional education and training [39-43]. Various strategies for implementing guidelines, including electronic guidelines, have been evaluated [44,45,80]. However, no strategies have been identified which, in all circumstances, will result in a successful implementation of new knowledge or in the desired behavioral changes [46,47]. A Health Technology Report (HTA) published in 2004 states that " [...] despite 30 years of research in this area, we still lack a robust generalisable evidence base to inform decisions about strategies to promote the introduction of guidelines or other evidence-based messages into practice" [48].

### Continuing medical education and knowledge translation

The German Society for General and Family Medicine (DEGAM) has identified general deficits in the professional training of GPs. A statement stresses: "From the point of view of general practitioners, the current situation in the field of further education is a discontenting one, characterized by outdated didactical methods, insufficient consideration of individual learning requirements, and an overemphasized focus on specialized disciplines as well as on the interests of the pharmaceutical industry" [49]. Worldwide there are efforts to improve the further education of GPs. Studies indicate that roughly one third of all changes in clinical practice are related to further education and training [50]. In this regard, a shift from "continuing medical education (CME)" to "continuing professional development (CPD)" can be detected [51-55]. Professional development is characterized by methodological variety and a diverse curriculum which attempts to consider the various preferences and types of learning. The methods also include the formative or summative assessment of acquired competences. "Stand-alone" CME programs seem to be less successful than programs with multi-modal interventions [47,56-58].

The "new media" are clearly gaining relevance within standard curricula. Electronic programs are being used for knowledge translation as well as knowledge assessment [59-63]. Data suggest that online-training may even result in a change of the GPs' behavior [62,64]. Problem-oriented learning via internet may also provide another promising option [65]. If students are accustomed to this form of learning, positive experiences made during the university education may be transferred to continuing education [66-68].

"Blended learning", which blends the features of classical or ex-cathedra teaching with those of e-learning, marks the threshold to a new concept of further education in the field of medicine [60,66,69,70]. "Blended learning is based on the recognition that a learning system based purely on electronic learning can only offer limited efficiency. Therefore blended learning combines e-learning with standard teaching methods or, rather, various teaching/learning media. Course content is conveyed face-to-face as well as via WBT (web based training), CD-Rom or print media. Learners are not bound to a specific medium. Instead they have the option of choosing a medium with suits their individual learning preferences. Thus blended learning involves the merging of e-learning/teaching with classical forms of learning/teaching, with the goal of providing an optimal overall concept" [71].

Strategies for applying the relatively new tools of e-learning to medicine have yet to be developed [46]. In order to do so, the needs of the users should first be analyzed. In addition, it should also be clarified what they want to learn and how they want to learn [8,38]. Suitable instruments need to be developed for this purpose. Only then we will be able to develop efficient (online) CME and CPD programs that implement sustainable, evidence-based guidelines respectively new knowledge and lead to an improvement of medical care. In this respect, a large need for research has been identified.

As a first step, it is important to clarify how doctors learn and make sure that their knowledge and skills are up-to-date. The following questions serve to determine this [40]:

(1) "Which educational interventions work in which situations?"

(2) "What factors promote the adoption of guidelines?"

(3) "Which factors inhibit their adoption?" (Authors note: barrier analysis)

(4) "What type of physician learning happens at what stages?"

The goal of the WIDA study is to compare the blended-learning concept with conventional training methods in order to assess whether the new concept can lead to an improved knowledge translation of dementia-related methods of diagnosis and treatment.

### Study goals

WIDA seeks to achieve the following goals:

• Creation of two different training concepts for transferring evidence-based knowledge on dementia to participants of GP quality circles (randomization on the level of the quality circles)

• Testing of contents and didactic materials with a large number of GPs in terms of knowledge gain and (to some extent) behavior-related changes

• Identification and description of

∘ content-related barriers, for example GPs' reluctance to deal with the dementia syndrome

∘ technical barriers, for example the chosen media or e-learning tools

The study results provide insights into how GPs can best be trained in an evidence-based and decentralized way.

## Methods/Design

The study involves a comparison of two dementia-related training concepts:

• Concept A consists of a structured case discussion study within the GPs' quality circle, moderated by a trainer. Participants are required to prepare independently beforehand by attending e-training sessions at a designated Internet site ("blended-learning" approach). Training "reminders" are regularly sent to increase the GPs' motivation.

• Concept B consists of a conventional face-to-face training in the GPs' quality circle with the same structured case discussion as in concept A ("classical" approach).

In order to detect possible changes in the general conditions (e.g. new guidelines of the health insurances, new financial regulations for GPs), the study also includes a control group consisting of other GPs who are given a print out of the dementia guidelines only.

## Hypotheses

It is assumed that the knowledge gain (KG) between t_0 _and t_1 _will be higher in concept A ("blended-learning" approach) than in concept B. However this hypothesis will be tested in a two-sided manner ("classical" approach).

### Primary and secondary outcomes of the WIDA trial

The study's primary outcome is to compare the effectiveness of two different types of interventions, with the goal of determining whether one of these concepts leads to more effective transfer of knowledge than the other.

Secondary outcome involve

• possible changes in the behavior of GPs after intervention

• utilization or non-utilization of e-learning tools (barrier analysis)

• the acceptance of new learning tools and methods in GPs' quality circles

### Selection of outcomes

Primary outcome is the knowledge gain between pre- and post-testing in the comparison of the two groups, assessed via two standardized questionnaires circulated at times t_0 _(before the training) and t_1 _(after the training).

Secondary outcomes regard both general knowledge gain (t_1 _and t_2_) and a comparison of the two groups at time t_2_. Possible behavioral changes on the part of GPs are ascertained with the help of four parameters taken from a sample of participants (t_2_) [4]. The information will be gleaned from the IT systems used in the GPs' offices:

• parameter 1: frequency of dementia screenings by psychometrical testing procedures in the medical practice, determined through EBM figures (EBM = *Einheitlicher Bewertungsmaßstab *= standardized assessment factor), stored in the accounting file

• parameter 2: frequency of diagnosis „dementia", determined through ICD codes

• parameter 3: frequency of prescription of selected anti-dementives (cholinesterase inhibitors, Memantine, Piracetam, Ginkgo-biloba compounds),

• parameter 4: characteristics of GPs' treatment, determined via EBM figures

Further steps are

• to assess use or non-use of additional e-learning tools in study arm A, measured via questionnaires at t_1 _and t_2_

• to qualitatively analyze discussions in the quality circles

• to qualitatively analyze interviews carried out in a sample group of participants from both training approaches.

### Schedule for training and evaluation

**Figure 1:**
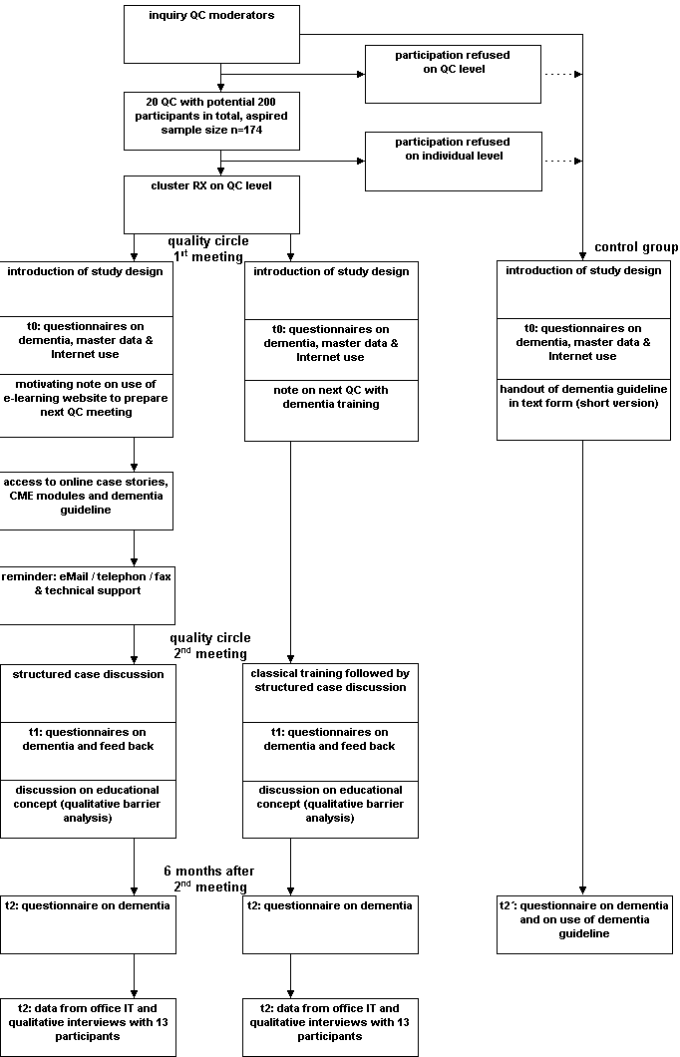
Flow chart training and evaluation.

A time period of three to six months is scheduled for recruiting GP quality circles and for attending two consecutive QC meetings (t_0 _and t_1_) (figure 1). Evaluation at time t_2 _is scheduled six months or two quarters (± 4 weeks) after the face-to-face training on dementia (t_1_). The evaluation and results can therefore be expected by late 2007.

### Specification of research questions

#### Study population and selection criteria

The planned training primarily addresses general practitioners, physicians, and specialists for internal medicine also working as GPs. Therefore, the WIDA study should orient its setting to the GP's environment as closely as possible. Quality circles will be recruited either by a letter to QC moderators signed by the head of the study or through personal contact. The plan is to recruit 174 GPs in approximately 20 quality circles in North-Rhine Westphalia (see estimation of number of cases). Willingness to participate is considered a prerequisite for participation. In the event that not enough GPs can be recruited, the search will be extended to other quality circles which may also be located in other regions or federal states.

#### Participation requirements

Participants in the study are required to participate in two consecutive QC meetings. In the event that a physician participates in more than one of the participating quality circles, s/he is enlisted at the time of his/her first contact with the study. An exchange with participants from other quality circles is theoretically possible, yet improbable. In the event that a participant is active in different circles, his/her participation is defined in terms of the circle where s/he was first introduced to the study.

Another requirement is the availability of Internet access (either in the medical practice or at home). GPs without internet access may participate, but their performance will not be included in the analysis. The following parameters will also be determined (table 1):

**Table 1:** Parameters to be ascertained

**Quality Circles (QC)**
Size of QC (estimated in advance by moderator)
Number of QC participants at t_0 _and t_1_
Number of QC participants actually participating in the study (t_0 _and t_1 _plus declaration of consent)
„Classical" Quality Circle or Practice Network
Interdisciplinary Quality Circle (yes/no)
Area of Medical Association (KVNO, KVWL)
Average number of QC meetings per year
Training on dementia/geriatric assessment within the past 6 or 12 months

**Participants**

Training on dementia/geriatric assessment within the past 6 or 12 months
Age
Sex
Internet user (yes/no*)
E-learning experience („Have you previously participated in a medical training via internet or CD-ROM?")

*excluding criterion

#### The interventions

After consent has been given by the QC moderators, all participating QCs will be visited at time t_0 _by one or several physicians working at the Competence Center for General Practice and Outpatients' Health Care, Witten/Herdecke-University. Having been informed of the study goals and the time required for participation in the WIDA study, the QC members will be invited to participate. After filling out a declaration of consent, the participants' master data are entered onto a data sheet. Next, all participating doctors receive a questionnaire to test their knowledge. The questionnaire contains 20 multiple choice questions on dementia. Internet access is also checked by means of a standardized questionnaire. All doctors in one participating QC are assigned to either group A or B in the first QC meeting (t_0_) (figure 1). An envelope containing the assignments will be opened by the facilitating doctor at the end of the introductory session; up to that time the envelope will remain closed.

Six months (± 4 weeks) after the intervention 13 participants from each group will be selected (t_2_). The aim is to gather relevant data for measuring a potential behavioral change by analyzing data from the doctor's IT. This data is then transferred to a structured data sheet. In addition, based on a semi-standardized check list, a qualitative interview is conducted, documented in digital form and transcribed later on (figure 1).

#### Specifics of study arm A

Having filled out the questionnaire (see above), participants are introduced to the e-learning tools (t_0_). They are given the relevant internet address (URL), and informed that a case discussion is scheduled for the next QC session which calls for their independent study before the meeting. The motivation to make use of the e-learning tools is considered a key factor for the success of training concept A.

Between the two training dates (t_0_-t_1_) all doctors participating in study arm A are provided with access to the WIDA's new internet site. There participants can find the latest evidence-based knowledge on dementia. The e-learning tools include:

• the electronic version of the dementia guideline – both in hypertext and pdf format

• two online case stories on dementia

• three testing modules on dementia allowing for the consecutive acquisition of CME credit points

• additional media for knowledge acquisition

Regular reminders as well as online support serve to increase the general acceptance and use of the e-learning tools. The technical aspects of the e-platform are described elsewhere [88].

##### Reminders

E-mail reminders can support the use of electronic guidelines and web-based training (Online-CME) [46,48,72]. In order to enhance participants' motivation to use the internet-based materials, training concept A makes use of a maximum of five e-mail reminders/telephone calls/letters (t_0 _- t_1_). Physicians receive the first reminder in the week after the first training-related QC event. Reminders can also be mailed in the form of a newsletter focusing on dementia. Alternatively, the reminder can be sent via telefax. All reminders follow a standard procedure, i.e. all participants of group A receive the same information.

##### Support

In addition to the planned reminders, participants are given an e-mail support address plus phone and fax number in order to receive timely technical support (t_0_-t_1_) (hotline installation). Discussion of further questions and feedback is reserved for the second QC meeting; thus the support does not include a content-related tutoring.

During the second QC meeting (t_1_), study arm A immediately starts with the structured case discussion. Following that, participants are once again asked to fill out the questionnaire on dementia-related questions (20 multiple choice questions). Simultaneously, the use or non-use of the e-learning tools is checked by means of a second questionnaire. Subsequently, there is an open discussion of the study, its educational content as well as its media.

Also, it is pointed out that participants will once again receive the questionnaire after another six months (by post). At the end of the second QC meeting, participants will be provided with a printed handout.

#### Specifics of study arm B

After having agreed to participate in the study, physicians learn that they will be trained on dementia in their next QC meeting. At this second meeting (t_1_), GPs receive a dementia-related training based on a slide presentation. The training will then be followed by a structured case discussion similar to the one described above for study arm A. This case discussion is also followed by the knowledge test with 20 multiple choice questions, a general discussion of the WIDA study and the reminder that the questionnaire will once again be sent to participants (by post) in six months time. At the end of this QC session, participants receive a printed handout.

#### Control group without intervention

Having given their consent to participate in an anonymous test of their knowledge, the QC participants are tested during the first QC meeting. Participants also receive a printed version of the dementia guideline. Six months later, participants again receive the questionnaire on dementia per regular mail along with a questionnaire pertaining to the frequency with which they used the guideline. Data is gathered for the control group only at t_0 _and t_2' _(figure 1).

#### Development of the learning material

In the WIDA study, the scientific framework for the GP training is an evidence-based guideline to the dementia syndrome provided by two authors (HCV, MB) of Witten/Herdecke University. The guideline has been updated three times since its first publication in March 2001 (last update: May 2005) [73]. It presents a condensed version of international documents and studies adapted to the German health care system [73]. The guideline also serves as a basis for the DEGAM dementia guideline which is presently being developed and approved.

In the context of the WIDA study adequate study materials (printed short form of the dementia guideline) are developed and given to the participants. Also, a special internet site is prepared for e-learning and containing online case stories, CME modules as well as the guideline in hypertext and pdf-format.

### Statistics

Chi-Square test is used to analyze dichotomous and categorical variables. Mann-Whitney U Test is used for comparing continuous variables in independent groups. A p-value <0.05 is considered to indicate a statistical significance.

The evaluation is carried out as follows: differences between the cumulative values of the pre-test questionnaire and those of the post-test questionnaire are determined. The distribution of these differences in each group is analyzed by means of a T-test for statistical relevance. Mean values and standard deviation from difference values are indicated. Additionally, a calculation via ANCOVA model is planned. Calculation is carried out according to an intention-to-treat (ITT) principle, i.e. all physicians are included in the analysis, even those who did not make use of the alternative learning methods. A subgroup analysis is planed for the participants of the blended learning approach according to frequency of their internet use.

### Randomization

Cluster randomization (RX) takes place at QC level. In order to attain the aspired base of 87 participants per group, we assume that it will be necessary to recruit 20 quality circles (see below). This should be a sufficient number for safeguarding the compatibility of both groups' participants (in terms of numbers, sex, age, etc). If necessary, more quality circles can be included in the study.

Stratified randomization was performed separately for small and large quality circles where the cut-off was chosen at 12 members per QC. Within each group block randomization with variable block size (max. 6 per block) was applied. Group allocation was then placed in sealed opaque envelopes with consecutive numbering for each stratum.

### Measures against confounding

From a methodological point of view, the largest problem of cluster-randomized studies is the risk of a selection bias [74]. Even if the allocation of quality circles to the two training groups is done randomly, physicians' inclusion or exclusion cannot be completely controlled. The exclusion of certain physicians might lead to structural inequality. Therefore, an *a posteriori *comparison of participants' basic data will be carried out in both training groups.

Further undesired effects might occur due to health care-related measures (such as campaigns). Therefore, a low-threshold intervention is carried out in about ten additional quality circles (distribution/mailing of guideline in printed form). Participants in these circles simultaneously receive the knowledge-testing questionnaire and another questionnaire concerning the use of the printed guideline. It has been suggested that merely passive strategies for disseminating and implementing new knowledge or new guidelines are largely ineffective [48]. However, the effort needed to study such an additional control group with low threshold intervention seems justified in order to map disturbance variables.

#### Consideration of drop-outs

Evaluation of the study's primary inquiry is done according to the ITT principle (see above), where all physicians are evaluated regardless of mode and degree of their actual participation in the training course to which they are primarily assigned. Withdrawal from the study is possible if the declaration of consent is revoked. In all cases, the data collected up to the point of exclusion or drop-out is evaluated.

### Reflections of outcome parameters

#### Primary outcome: knowledge gain

Knowledge gain is measured by employing a multiple choice questionnaire on two levels:

1. It was first used in a pilot test of ten physicians participating in the Witten/Herdecke University's quality circle.

2. After revising the 20 items with 5 characteristics each, the questionnaire was used in the context of the "Dementia in General Medicine" (IDA) initiative by the AOK Bavaria between August and November 2005 [75,76]. In this project, 132 GPs were trained in Bavaria and the questionnaire was subsequently employed. Here, a significant knowledge gain of 4.0 ± 2.6 questions (confidence interval 3.6 – 4.5, p < 0.001) was identified. The comparison of two different training groups displayed a difference of mean values of 3.1 ± 2.1 (p < 0.001). In both cases, this resulted in an effect size of 1.5 (Cohen's d) [76]. The thus evaluated questionnaire will be used in the WIDA study. However, an effect size of 1.5 appears to be too optimistic. A current US study compared an online training with a classical face-to-face training and assumed an effect size of 0.75 [62]. Extensive investigation did not identify directly comparable research on the effects of a blended-learning concept which could have served as a basis for sample size calculation. Therefore the WIDA study assumes a conservatively assessed medium effect size of 0.5. Using this effect size, and assuming standard error assumptions (α = 5%, power = 80%). The target sample size is 64 per group, or 128 in total (G-Power software).

This sample size allows us to check whether the two training concepts differ by about 0.5 SD which corresponds to about one (or more) correctly answered question. In case of a non significant result we could be about 80% certain (power) that the difference between both approaches is not more than one correct question. This means that both approaches are roughly comparable (non-inferiority) while approach A (blended-learning) will maybe require fewer resources.

#### Consideration of the design effect and calculation of the drop-out rate

The so-called design effect (DE) is assessed according to: DE = 1 + ICC (m-1) with ICC matching the intra-cluster correlation coefficient m of the average cluster size. The average cluster size (corresponding to the average number of participants in a QC) is assumed to be 10. For the ICC, values between 0.01 and 0.02 for studies on patients are given in the literature [82]. For intervention studies on physicians, the assumed values lie mostly between 0.02 and 0.1 [81,83,84]. With our purposes, a value of 0.04 is assumed. This results in a design effect of: 1 + 0.04 (10-1) = 1.36.

Thus the sample size assessment given above (n = 128) should be multiplied by this factor. This results in a sample size of 174 test persons.

It is furthermore assumed that approximately 15% of QC participants will refuse participation or will not show up due to schedule conflicts. Therefore, to achieve the desired sample size of 174, 20 quality circles with an average of 10 participants or a total of 200 physicians are necessary.

#### Secondary outcomes: behavior modification

An informal interviewing of doctors teaching at Witten/Herdecke University revealed that they were extremely reluctant to provide patient data for research purposes. It must be assumed that GPs without a university affiliation will show an ever greater reluctance to provide data from their medical practice. Therefore, we have refrained from considering changes in prescription behavior as a primary goal parameter. Based on the rather low prevalence (an estimated 12 dementia patients per medical practice per quarter with a very broad distribution, see below) it also does not seem feasible – due to limited duration of study and resources – to collect patient data directly for the purpose of comparing the effectiveness of the two training concepts. Yet, in order to receive at least an indirect impression of what happens at the level of GPs' behavior, an attempt is made to visit 13 medical practices (= 15% of all participants) per group. The reason for doing this is

1. to extract treatment-relevant data from the physicians files

2. to conduct qualitative interviews with GPs.

The 13 control samples are selected from each group according to their informed consent. In the event that a GP refuses further participation at this point, the attempt is made to find out why. Another participating GP is then selected.

The evaluation of the behavioral modification parameters is based on the following assumptions:

• Average number of patients per GP practice per quarter = 1000 (data provided by the Medical Association of Westfalen-Lippe as of September 20, 2005).

• Average portion of retirees in GPs' practices: 35% (data provided by the Medical Association of Westfalen-Lippe, as of September 20, 2005).

• Prevalence of dementia in Germany in the 65+ age group: 7.2%, equivalent to nearly one million patients [12].

• Incidence of dementia in Germany in the 65+ age group: 1.9%, equivalent to approximately 230,000 incidences per annum [12].

• Roughly 83,000 patients received anti-dementives whose use is considered evidence-based (as of 2003) [77]. This means that about 8.3% of all dementia patients received recommended medication. This corresponds to information given in literature which also states that the prescription frequency of recommended anti-dementives is 9% for Germany [4,20]. (Note: This does not suggest that one million dementia patients should be treated with anti-dementives. As a matter of course, a very tight medical indication and existing contra-indications imply that only a fraction of dementia patients can be considered for drug therapy.)

• About 440,000 patients have been treated with anti-dementives which must be considered questionable with respect to effectiveness (as of 2003) [77]. This corresponds to 44% of all dementia patients.

• The invoicing of patients covered by compulsory health insurance, is based on the currently valid standardized assessment factor (EBM 2000plus), which considers revisions made by the assessment commission (valid as of April 1, 2005) as well as revisions of § 87, paragraph 3, SGB V (effective as of July 1, 2005).

• The billing of patients covered by a private health insurance is based on the January 2002 tariff for physicians (GOÄ).

The analysis of behavior modification data is carried out as follows:

• Summarizing of collected data (t_2_) from participating medical practices of groups A and B in the four quarters prior to intervention; then generation of the arithmetic average. The average of four quarters is used in order to limit possible distortion (e.g. due to the introduction of EBM 2000plus).

• Summarizing of collected data (t_2_) from all participating practices of groups A and B in the two quarters after intervention and generation of the arithmetic average. Due to the maximum duration of the study, only two quarters are averaged.

• Comparison of average values.

• Comparison with the data provided by the Medical Association of Westfalen-Lippe to the extent that it is available for the period of reference.

#### Addenda: screening

Based on an average of 350 patients over 65 years per quarter, a dementia prevalence of 7.2% in the overall population and assuming that only 50% of all dementia patients undergo GP treatment, we calculate an average of 12 dementia patients per GP. In spite of indications pointing to a possibly higher prevalence in GP practices [33,78], this estimate is intentionally a conservative one. A survey of British GPs reveal that psychometric tests are used in only 15% of all cases in which the patient is believed to be suffering from dementia [79]. Because this data is missing for Germany, we use these 15% as a reference value. Assuming an average of per GP and quarter, it is therefore estimated that a screening is conducted on only two patients. An increase in screening can be expected after dementia training.

#### Addenda: prescription of anti-dementives

With 12 dementia patients per quarter, approximately 8% or one patient would receive a recommended drug while 44% (nearly 5 patients) would receive a non-recommended anti-dementive [77].

In order to compare prescription frequency of anti-dementives before and after the training sessions, a quotient is created and compared; the expectation is that the quotient will increase:

Aprä+Aprä−<Apost+Apost−
 MathType@MTEF@5@5@+=feaafiart1ev1aaatCvAUfKttLearuWrP9MDH5MBPbIqV92AaeXatLxBI9gBaebbnrfifHhDYfgasaacH8akY=wiFfYdH8Gipec8Eeeu0xXdbba9frFj0=OqFfea0dXdd9vqai=hGuQ8kuc9pgc9s8qqaq=dirpe0xb9q8qiLsFr0=vr0=vr0dc8meaabaqaciaacaGaaeqabaqabeGadaaakeaadaWcaaqaaiabbgeabnaaBaaaleaacqqGWbaCcqqGYbGCcqqGKda5aeqaaOGaey4kaScabaGaeeyqae0aaSbaaSqaaiabbchaWjabbkhaYjabbsoaKdqabaGccqGHsislaaGaeyipaWZaaSaaaeaacqqGbbqqdaWgaaWcbaGaeeiCaaNaee4Ba8Maee4CamNaeeiDaqhabeaakiabgUcaRaqaaiabbgeabnaaBaaaleaacqqGWbaCcqqGVbWBcqqGZbWCcqqG0baDaeqaaOGaeyOeI0caaaaa@4BFC@

Aprä + : recommended anti-dementives prior to intervention

Aprä - : non-recommended anti-dementives prior to intervention

Apost + : recommended anti-dementives following intervention

Apost - : non-recommended anti-dementives following intervention

#### Addenda: Qualitative analysis

Qualitative interviews with 13 participating physicians per group take place at t_2_. Table 2 presents a "correlation" of quantitative and qualitative parameters.

**Table 2:** Dimensions of knowledge translation (knowledge gain; behavior modification)

**Quantitative parameters**	**Qualitative parameters (following a structured interview guideline)**
number of participants, response of questionnaires, use of training measures	• questions regarding user behavior • identification of barriers

knowledge gain measured by two questionnaires (pre- and post test)	• subjective evaluation of knowledge gain

"behavior modification screening" gathered by EBM2000plus (GOÄ):	
• (03313 orientation survey of psychopathological status) • 03314 (857) mental tests • 03341 GP-geriatric basic assessment investigation at t_2_: assessment of 4 quarters before and 2 quarters after intervention	• self-evaluation of behavior • identification of barriers

„behavior modification diagnosis/differential diagnosis" assessed via six figures of ICD-10-GM 2005: F00, F01, F02, F03, F07, G32, R54 data gathered at t_2_: assessment of 4 quarters before and 2 quarters after intervention	• question whether diagnostic behavior has changed • identification of barriers

„behavior modification therapy" ascertained via prescriptions of anti-dementives*:	
• cholinesterase inhibitors • memantine • ginkgo biloba compounds • piracetam investigation at t_2_: assessment of 4 quarters before and 2 quarters after intervention	• identify whether there is an increase in the prescription of evaluated anti-dementives (e.g. cholinesterase inhibitors, memantines) and whether there is a decrease in the prescription of untested or unreliable anti-dementives (e.g. ginkgo biloba compounds, piracetam) • question regarding future drug therapy • identification of barriers

„behavior modification treatment" gathered by EBM2000plus: 03001 coordination of GP-related care 03002 coordination of GP-related care in nursing homes 21230 continuous co-care in the domestic environment 21231 continuous co-care in a nursing facility 21232 psychiatric treatment investigation at t_2_: assessment of 4 quarters before and 2 quarters after intervention	• question regarding prescription of non drug-related therapy • question regarding support groups for family members • interaction with patients and family members when patient is suspected to suffer from dementia and when the diagnosis has been confirmed • identification of barriers

* Based on the most frequent prescriptions in Germany 2003 [77]

#### Addenda: Internet use

Internet use is ascertained anonymously with the help of a "counter" and questionnaires administered at t_1 _and t_2_. A personalized tracking of internet users was not performed:

1. for reasons of privacy and data protection (see data protection)

2. because the recruitment of GPs in other projects revealed that personalized tracking would be viewed apprehensively and lead to a massive reduction in the number of participants or a rejection of e-learning tools.

For this reason there is no personalized tracking of participating GPs. Instead, a counter at the study's website registers the number of site visits. It is assumed that third parties will not use the WIDA site since URL information will be restricted to participants until completion of the study. In addition to user data collected in this manner, participants will be asked about length and intensity of their e-training sessions in the questionnaire. In this way a correlation between duration of use and outcome can be expected.

### Data management

#### Data collection

Collection of GP data at the time of the study's inception (t_0_) and at t_1 _and t_2 _is per hard copy. Postage-paid envelopes are provided so that the GPs will send back the questionnaire at t_2_. Selected medical practices send their data, which are identified via differentiated search strategies and transferred via BDT-interface in anonymous form, to a storage medium. In the event that this is technically not possible, the office staff provides the necessary data by filling out a form. Office personnel are briefed by UWH employees in advance with respect to the type of data required. This ensures that patient files cannot be accessed by UHW employees.

#### Scheduling of data collection

Various methods of data collection are used in this study. Questionnaires are filled out by the participating physicians. There is also an evaluation of data from the web-server which provides for online learning.

The WIDA study foresees the following investigation schedule:

• t_0_: acceptance of GPs to the study (first study-related QC meeting)

• between t_0 _and t_1_: data on internet use (group A only)

• t_1_: after approximately three months (second study-related QC meeting)

• t_2_: 6 months (± 4 weeks) after t_1 _(control group: t_2'_)

The amount of documentation required by GPs is kept low so as not to jeopardize the participants' motivation.

#### Data protection

For the various phases of processing patient-related data (storage, modification, transfer, deletion and use) and data security, the valid directives of the Federal Data Security Act (*Bundesdatenschutzgesetz, BDSG*) and the various special regulations (e.g. SGB V, SGB VII, SGB IX) for data processing shall be complied with and the necessary technical and organizational measures taken. § 40 – "processing and use of person-related data by research institutions" – clause 2 of the Federal Data Protection Act (*BDSG*) states:

„Person-related data must be rendered anonymous as soon as the research purpose allows for it. Up to this point, any attributes which make it possible to assign particular personal or factual circumstances to a certain or ascertainable person must be stored separately. Such attributes may only be assigned to their owner in as much as necessitated by the research purpose."

Accordingly all research-relevant data is saved anonymously. Person-related data is kept separately only if this data is required for establishing contact and for correspondence.

#### Premature termination of the study

Due to close-meshed monitoring, recruiting problems or problems of data quality can be detected at an early stage. It is therefore possible to take appropriate counteractive measures. For this reason premature termination of the study due to insufficient recruitment is unlikely. The same holds true for premature termination due to new scientific insights. Moreover, premature termination due to early results regarding the superiority of one training concept is also not likely since training will be completed long before first trends will become recognizable. In the event that the study must be terminated prematurely due to unforeseeable reasons, the data collected up to that point will nonetheless be published. Any decision regarding a possible premature termination of the study lies in the responsibility of the investigators in consultation with the Federal Ministry of Education and Research (*BMBF*).

## Competing interests

None of the involved persons has claimed to have a conflict of interests. More particularly, there are no financial or personal relations or dependences to other persons or organizations which might restrict the autonomy of activities.

The study will be carried out according to the guidelines of the study manual and the standards of "good epidemiological practice (GEP)". The study trial is registered in an international register (to take place under ISRCTN36550981) and has a positive vote of the responsible ethics commission (Nr. 42/2006). The study's results will be published independently of the study's outcome.

## Authors' contributions

HCV conceived and developed this study and drafted the manuscript. He collects and collates the data and will assist with statistical analysis. MB helped to design the study and drafted the manuscript. RL also assisted in the study design, will perform the statistical analysis, formulated the randomization and helped to draft the manuscript. MR helped to design the study, will assist with statistical analysis and drafted the manuscript. All authors read and approved the final manuscript.

## Sources of funding

With regard to its primary endpoint „knowledge gain", the study is funded by the Federal Ministry of Education and Research (*BMBF*) via the German Aerospace Center *(DLR*) under project number 01GK0512. The applicants intend to obtain auxiliary third-party funds for further investigations (especially with regard to the secondary outcomes). In no case participating scientists will receive any personal third-party financial support.

## Pre-publication history

The pre-publication history for this paper can be accessed here:



## References

[B1] Seufert S, Mayr P (2002). Fachlexikon e-le@rning.

[B2] Hallauer JF, Kurz A (2002). Weißbuch Demenz.

[B3] Melchinger H, Machleidt W (2005). Werden Demenzpatienten in Hausarztpraxen lege artis behandelt? Ergebnisse einer Interviewstudie. Zeitschrift für Allgemeinmedizin.

[B4] Melchinger H, Machleidt W (2005). Hausärztliche Versorgung von Demenzkranken. Analyse der Ist-Situation und Ansätze für Qualifizierungsmaßnahmen. Nervenheilkunde.

[B5] Bundesministerium für Familie Senioren Frauen und Jugend (2002). Vierter Altenbericht zur Lage der älteren Generation in der Bundesrepublik Deutschland: Risiken, Lebensqualität und Versorgung Hochaltriger – unter besonderer Berücksichtigung demenzieller Erkrankungen.

[B6] van Hout H, Vernooij-Dassen M, Bakker K, Blom M, Grol R (2000). General practitioners on dementia: tasks, practices and obstacles. Patient Educ Couns.

[B7] Wagner G, Abholz H (2002). Diagnose und Therapiemanagement der Demenz in der Hausarztpraxis. Zeitschrift für Allgemeinmedizin.

[B8] Kaduszkiewicz H, van den Bussche H (2003). Die hausärztliche Versorgung von Patienten mit kognitiven Störungen und Demenz. Psychoneuro.

[B9] Kaduszkiewicz H, Sperber S, van den Busche H (2005). Kompetenz bei Demenz. Möglichkeiten und Grenzen der hausärztlichen Versorgung von Patienten mit kognitiven Störungen und Demenzen. Niedersächsisches Ärzteblatt.

[B10] Bickel H, H. F (2002). Epidemiologie psychischer Störungen im Alter. Lehrbuch der Gerontopsychiatrie und -psychotherapie.

[B11] Bickel H (2001). [Dementia in advanced age: estimating incidence and health care costs]. Z Gerontol Geriatr.

[B12] Bickel H (2000). [Dementia syndrome and Alzheimer disease: an assessment of morbidity and annual incidence in Germany]. Gesundheitswesen.

[B13] Jost BC, Grossberg GT (1995). The natural history of Alzheimer's disease: a brain bank study. J Am Geriatr Soc.

[B14] Larson EB, Shadlen MF, Wang L, McCormick WC, Bowen JD, Teri L, Kukull WA (2004). Survival after initial diagnosis of Alzheimer disease. Ann Intern Med.

[B15] Wolfson C, Wolfson DB, Asgharian M, M'Lan CE, Ostbye T, Rockwood K, Hogan DB (2001). A reevaluation of the duration of survival after the onset of dementia. N Engl J Med.

[B16] Ueki A, Shinjo H, Shimode H, Nakajima T, Morita Y (2001). Factors associated with mortality in patients with early-onset Alzheimer's disease: a five-year longitudinal study. Int J Geriatr Psychiatry.

[B17] BDA, Berufsverband der Allgemeinärzte (1999). Manual Demenz.

[B18] Hallauer JF, Schons M, Smala A, Berger K (2000). Untersuchung von Krankheitskosten bei Patienten mit Alzheimer-Erkrankung in Deutschland. Gesundheitsökonomie & Qualitätsmanagement.

[B19] Carlson MC, Tschanz JT, Norton MC, Welsh-Bohmer K, Martin BK, Breitner JC (2002). H2 histamine receptor blockade in the treatment of Alzheimer disease: a randomized, double-blind, placebo-controlled trial of nizatidine. Alzheimer Dis Assoc Disord.

[B20] Ruof J, Mittendorf T, Pirk O, von der Schulenburg JM (2002). Diffusion of innovations: treatment of Alzheimer's disease in Germany. Health Policy.

[B21] O'Connor DW, Fertig A, Grande MJ, Hyde JB, Perry JR, Roland MO, Silverman JD, Wraight SK (1993). Dementia in general practice: the practical consequences of a more positive approach to diagnosis. Br J Gen Pract.

[B22] Eefsting JA, Boersma F, Van den Brink W, Van Tilburg W (1996). Differences in prevalence of dementia based on community survey and general practitioner recognition. Psychol Med.

[B23] Biernat K, Simpson D, Duthie E, Bragg D, London R (2003). Primary care residents self assessment skills in dementia. Adv Health Sci Educ Theory Pract.

[B24] Waldorff FB, Almind G, Makela M, Moller S, Waldemar G (2003). Implementation of a clinical dementia guideline. A controlled study on the effect of a multifaceted strategy. Scand J Prim Health Care.

[B25] Cooper B, Bickel H, Schaufele M (1992). The ability of general practitioners to detect dementia and cognitive impairment in their elderly patients. A study in Mannheim. International Journal of Geriatric Psychiatry.

[B26] Dwolatzky T, Clarfield AM (2004). Assessement of dementia in primary care setting. Expert Review of Neurotherapeutics.

[B27] Iliffe S, Mitchley S, Gould M, Haines A (1994). Evaluation of the use of brief screening instruments for dementia, depression and problem drinking among elderly people in general practice. Br J Gen Pract.

[B28] Mant A, Eyland EA, Pond DC, Saunders NA, Chancellor AH (1988). Recognition of dementia in general practice: comparison of general practitioners' opinions with assessments using the mini-mental state examination and the Blessed dementia rating scale. Fam Pract.

[B29] Pentzek M, Abholz HH (2004). Das Erkennen von Demenzen in der Hausarztpraxis - eine kritische Übersicht zur Studienlage. NeuroGer.

[B30] Riedel-Heller SG, Schork A, Fromm N, Angermeyer MC (2000). [Dementia patients in general practice--results of a survey]. Z Gerontol Geriatr.

[B31] Waldorff FB, Moller S (2001). Diagnostic evaluation of dementia in general practice in Denmark. A national survey. Scand J Prim Health Care.

[B32] Wind AW, Schellevis FG, Van Staveren G, Scholten RP, Jonker C, Van Eijk JT (1997). Limitations of the Mini-Mental State Examination in diagnosing dementia in general practice. Int J Geriatr Psychiatry.

[B33] Pentzek M, Fuchs A, Abholz HH (2005). [The attitudes of General Practitioners regarding dementia - Cognitive, affective, and external components]. Nervenheilkunde.

[B34] Renshaw J, Scurfield P, Cloke L, Orrell M (2001). General practitioners' views on the early diagnosis of dementia. Br J Gen Pract.

[B35] Bowers J, Jorm AF, Henderson S, Harris P (1992). General practitioners' reported knowledge about depression and dementia in elderly patients. Aust N Z J Psychiatry.

[B36] Sandholzer H, Breull A, Fischer GC (1999). [Early diagnosis and early treatment of cognitive disorders: a study of geriatric screening of an unselected patient population in general practice]. Z Gerontol Geriatr.

[B37] Field MJ, Lohr KM (1992). Guidelines for clinical practice: from development to use.

[B38] Ärztliches Zentrum für Qualität in der Medizin (ÄZQ), AWMF (2005). Deutsches Instrument zur methodischen Leitlinien-Bewertung (DELBI).

[B39] Butzlaff M, Lutz G, Falck-Ytter C (1998). Lernen ohne Ende. Die medizinische Leitlinie - ein Weiterbildungsinstrument mit Zukunft?. Dtsch Med Wochenschr.

[B40] Davis D (2000). Clinical practice guidelines and the translation of knowledge: the science of continuing medical education. Cmaj.

[B41] Ollenschlaeger G, Kirchner H, Fiene M (2001). Leitlinien in der praktischen Medizin - scheitern sie an der Umsetzung. Internist (Berl).

[B42] Vollmar HC, Koneczny N, Floer B, Isfort J, Kunstmann W, Butzlaff M (2002). Evidenzbasierte Leitlinien als Instrumente des Wissenstransfers in die Praxis. Die Arbeitsweise des medizinischen Wissensnetzwerks evidence.de. Fortschr Med.

[B43] Vollmar HC, Kirchner H, Koneczny N, Engelbrecht J, Kunstmann W, Schürer-Maly CC, Löscher S, L. H, Butzlaff M, Ollenschlaeger G (2004). Online-Fortbildung: Realitätsnah lernen. Deutsches Ärzteblatt.

[B44] Bell DS, Fonarow GC, Hays RD, Mangione CM (2000). Self-study from web-based and printed guideline materials. A randomized, controlled trial among resident physicians. Ann Intern Med.

[B45] Butzlaff M, Vollmar HC, Floer B, Koneczny N, Isfort J, Lange S (2004). Learning with computerized guidelines in general practice?: A randomized controlled trial. Fam Pract.

[B46] Grol R, Grimshaw J (2003). From best evidence to best practice: effective implementation of change in patients' care. Lancet.

[B47] Oxman AD, Thomson MA, Davis DA, Haynes RB (1995). No magic bullets: a systematic review of 102 trials of interventions to improve professional practice. Cmaj.

[B48] Grimshaw J, Thomas R, MacLennan G, al. (2004). Effectivenness and efficiency of guideline dissemination and implementation strategies. Health Technol Assess.

[B49] Donner-Banzhoff N Professionelles Lernen - ein Leben lang. Stellungnahme zur ärztlichen Fortbildung.. http://www.degam.de.

[B50] Allery LA, Owen PA, Robling MR (1997). Why general practitioners and consultants change their clinical practice: a critical incident study. Bmj.

[B51] Cantillon P, Jones R (1999). Does continuing medical education in general practice make a difference?. Bmj.

[B52] Burrows P (2003). Continuing professional development: filling the gap between learning needs and learning experience. Education for Primary Care.

[B53] Charlton R (2001). Continuing professional development (CPD) and training. BMJ Classified.

[B54] Davis D, Evans M, Jadad A, Perrier L, Rath D, Ryan D, Sibbald G, Straus S, Rappolt S, Wowk M, Zwarenstein M (2003). The case for knowledge translation: shortening the journey from evidence to effect. Bmj.

[B55] Davis D, Goldman J, Perrier L, Dent JA, Harden RM (2005). Effective continuing professional developement. A practical guide for medical teachers.

[B56] Grant J, Chambers E, Jackson G (1999). The good CPD guide. A practical guide to manged CPD.

[B57] Davis D, O'Brien MA, Freemantle N, Wolf FM, Mazmanian P, Taylor-Vaisey A (1999). Impact of formal continuing medical education: do conferences, workshops, rounds, and other traditional continuing education activities change physician behavior or health care outcomes?. Jama.

[B58] Finkel SI, Lyons JS, Anderson RL, Sherrell K, Davis J, Cohen-Mansfield J, Schwartz A, Gandy J, Schneider L (1995). A randomized, placebo-controlled trial of thiothixene in agitated, demented nursing home patients. International Journal of Geriatric Psychiatry.

[B59] Cantillon P, Irish B, Sales D (2004). Using computers for assessment in medicine. Bmj.

[B60] Karnath BM, Das Carlo M, Holden MD (2004). A comparison of faculty-led small group learning in combination with computer-based instruction versus computer-based instruction alone on identifying simulated pulmonary sounds. Teach Learn Med.

[B61] Roex A, Degryse J (2004). A computerized adaptive knowledge test as an assessment tool in general practice: a pilot study. Med Teach.

[B62] Fordis M, King JE, Ballantyne CM, Jones PH, Schneider KH, Spann SJ, Greenberg SB, Greisinger AJ (2005). Comparison of the instructional efficacy of Internet-based CME with live interactive CME workshops: a randomized controlled trial. Jama.

[B63] Sandars J (2005). Using web quests to enhance work based learning. Work Based Learning in Primary Care.

[B64] Harris JM, Kutob RM, Surprenant ZJ, Maiuro RD, Delate TA (2002). Can Internet-based education improve physician confidence in dealing with domestic violence?. Fam Med.

[B65] Baehring TU, Fischer MR (1998). Problemorientiertes Lehren und Lernen in der Medizin: Neue technische und didaktische Möglichkeiten durch das WWW.. Biomedical Journal.

[B66] Taradi SK, Taradi M, Radic K, Pokrajac N (2005). Blending problem-based learning with Web technology positively impacts student learning outcomes in acid-base physiology. Adv Physiol Educ.

[B67] Zumbach J, Reimann P, Thissen F (2003). Computerunterstütztes fallbasiertes Lernen: Goal-Based Scenarios und Problem-Based Learnig. Multimedia-Didaktik in Wirtschaft, Schule und Hochschule.

[B68] Smits PB, Verbeek JH, de Buisonje CD (2002). Problem based learning in continuing medical education: a review of controlled evaluation studies. Bmj.

[B69] Shaffer K, Small JE (2004). Blended learning in medical education: use of an integrated approach with web-based small group modules and didactic instruction for teaching radiologic anatomy. Acad Radiol.

[B70] Gold JP, Begg WB, Fullerton D, Mathisen D, Olinger G, Orringer M, Verrier E (2004). Successful implementation of a novel internet hybrid surgery curriculum: the early phase outcome of thoracic surgery prerequisite curriculum e-learning project. Ann Surg.

[B71] Sauter AM, Sauter W, Bender H (2004). Blended Learning. Effiziente Integration von E-Learning und Präsenztraining.

[B72] Abdolrasulnia M, Collins BC, Casebeer L, Wall T, Spettell C, Ray MN, Weissman NW, Allison JJ (2004). Using email reminders to engage physicians in an Internet-based CME intervention. BMC Med Educ.

[B73] Vollmar HC, Koch M, Löscher S, Butzlaff M Demenz. Evidenzbasierte Leitlinie zu Diagnose und Therapie.

[B74] Puffer S, Torgerson D, Watson J (2003). Evidence for risk of bias in cluster randomised trials: review of recent trials published in three general medical journals. Bmj.

[B75] Lauterberg J, Großfeld-Schmitz M, Ruckdäschel S, Neubauer S, Mehlig H, Gaudig M, Hruschka D, Gräßel E, Vollmar HC, Holle R (2007). Projekt IDA – Konzept und Umsetzung einer cluster-randomisierten Studie zur Demenzversorgung im hausärztlichen Bereich. Z Arztl Fortbild Qualitatssich.

[B76] Vollmar HC, Gräßel E, Lauterberg J, Neubauer S, Großfeld-Schmitz M, Koneczny N, Schürer-Maly CC, Koch M, Ehlert N, Holle R, Rieger MA, Butzlaff M (2007). Multimodale Schulung von Hausärzten - Evaluation und Wissenszuwachs im Rahmen der Initiative Demenzversorgung in der Allgemeinmedizin (IDA). Z Arztl Fortbild Qualitatssich.

[B77] Schwabe U, Schwabe U, Paffrath D (2004). Antidementiva. Arzneiverordnungsreport 2004.

[B78] Arbeitskreis Demenz im RPL - Regionales Praxisnetz Leverkusen e.V. (2002). Demenz-Früherkennung: Pilotstudie aus einem Praxisnetz. Zeitschrift für Allgemeinmedizin.

[B79] Downs M, Cook A, Rae C, Collins KE (2000). Caring for patients with dementia: the GP perspective. Aging Ment Health.

[B80] Downs M, Turner S, Bryans M, Wilcock J, Keady J, Levin E, O'Carroll R, Howie K, Iliffe S (2006). Effectiveness of educational interventions in improving detection and management of dementia in primary care: cluster randomised controlled study. Bmj.

[B81] Kerry SM, Bland JM (1998). The intracluster correlation coefficient in cluster randomisation. Bmj.

[B82] Killip S, Mahfoud Z, Pearce K (2004). What is an intracluster correlation coefficient? Crucial concepts for primary care researchers. Ann Fam Med.

[B83] Rosemann T, Korner T, Wensing M, Gensichen J, Muth C, Joos S, Szecsenyi J (2005). Rationale, design and conduct of a comprehensive evaluation of a primary care based intervention to improve the quality of life of osteoarthritis patients. The PraxArt-project: a cluster randomized controlled trial [ISRCTN87252339]. BMC Public Health.

[B84] Wright FC, Law CH, Last LD, Klar N, Ryan DP, Smith AJ (2006). A blended knowledge translation initiative to improve colorectal cancer staging [ISRCTN56824239]. BMC Health Serv Res.

[B85] Cook DA (2005). The research we still are not doing: an agenda for the study of computer-based learning. Acad Med.

[B86] Wall TC, Huq Mian MA, Ray MN, Casebeer L, Collins BC, Kiefe CI, Weissmann N, Allison JJ (2005). Improving Physician Performance Through Internet-Based Interventions: Who Will Participate?. Journal of medical Internet research.

[B87] Mamary EM, Charles P (2000). On-site to on-line: barriers to the use of computers for continuing education. J Contin Educ Health Prof.

[B88] Vollmar HC, Schurer-Maly CC, Frahne J, Lelgemann M, Butzlaff M (2006). An e-learning platform for guideline implementation--evidence- and case-based knowledge translation via the Internet. Methods Inf Med.

